# Experiences of primary healthcare staff in Europe when caring for asylum seekers and refugees: A scoping review protocol.

**DOI:** 10.12688/hrbopenres.14413.1

**Published:** 2026-04-16

**Authors:** Kylie Fenech, Chiara Pittalis, Éidín Ní Shé, Gintare Valentelyte, Jakub Gajewski, Mary Rose Sweeney

**Affiliations:** 1 Faculty of Nursing and Midwifery, Royal College of Surgeons in Ireland, Dublin, Leinster, Ireland; 2 Institute of Global Surgery, School of Population Health, Royal College of Surgeons in Ireland, Dublin, Leinster, Ireland; 3 Graduate School of Healthcare Management, Royal College of Surgeons in Ireland, Dublin, Leinster, Ireland; 4 School of Population Health, Royal College of Surgeons in Ireland, Dublin, Leinster, Ireland

**Keywords:** Healthcare professionals, Healthcare providers, Staff experiences, Primary care, Community-based care, Asylum seekers, Refugees

## Abstract

**Background:**

Migration significantly influences the health of asylum seekers and refugees, as it heightens vulnerabilities and poor health. Health systems struggle with providing specific care to these populations due to specific health needs compounded by language, cultural and literacy barriers. Primary care offers critical opportunities for healthcare providers to address specific health inequities that asylum seekers and refugees face. Understanding provider experiences is essential to determine how health systems support their staff, caring for asylum seeking and refugee populations.

**Objective:**

This review will investigate the experiences of primary and community healthcare providers in the European Union (EU), providing care to asylum seekers and refugees, and the factors which influence these experiences.

**Method:**

The scoping review will follow the methodology from the Joanna Briggs Institute (JBI), alongside Arksey & O’Malley’s framework. The primary research question asks, “What are the experiences of healthcare staff while providing care to asylum seekers and refugees in primary and community settings?”. Secondary questions will explore contextual differences within the EU. Studies published between 2015 to 2025 will be searched across PubMed, MEDLINE, CINAHL, Embase, Scopus, PsycINFO and BASE databases, alongside grey literature searches. Data extraction will include information on the key aspects that shape experiences alongside potential challenges that have been documented. Comparisons will be made on the differences observed across professional roles and national contexts. Stakeholders have been consulted and will be presented with the findings for their interpretation into relevance and applicability across various contexts.

**Conclusion:**

This review aims to inform policy and practice by identifying key factors shaping healthcare staff experiences, thereby guiding improvements in primary care and migrant health services. This review will examine the experiences of community-based healthcare providers caring for asylum seekers and refugees, across the EU, with the aim of identifying the key factors that shape these experiences.

## Introduction

Migration significantly influences the health of individuals as they often face unique and distinctive risks before, during, and after re-settlement. ^1, 2^ Health outcomes frequently worsen due to the compounding effects of stressful migration journeys, inadequate living conditions, and limited healthcare access. ^1– 3^ Following re-settlement, asylum seekers and refugees (see Box 1) ^4, 5^ often encounter further challenges related to socioeconomic hardships, social isolation, and difficulties navigating unfamiliar healthcare systems. ^2, 3^

Box 1. **Definitions used for the terms ‘asylum seeker(/s)’ and ‘refugee(/s)’.**

An asylum seeker is a stateless individual has applied for protection under the Geneva Refugee Convention and Protocol within an EU member country but is awaiting a final decision on such. ^7^

A refugee on the other hand is a person who, owing to a well-founded fear of persecution for reasons such as race, religion, nationality, political opinion, or membership of a particular social group, is outside their country of nationality and cannot or will not seek its protection, and by definition someone whose asylum claim has been granted. ^8^

Such challenges may further exacerbate poor health issues as asylum seekers and refugees experience further disadvantage in an already strained health system. ^3, 6, 7^ Their health needs may be substantial, ^7^ necessitating a proactive and coordinated response from health systems to ensure positive health outcomes for this population. ^3^ Health systems may not be adequately equipped, resourced or prepared to deal with complex health needs, thus further compounding health inequalities. ^3, 7, 8^ Evidence shows that asylum seekers and refugees report worse health outcomes in high-income countries on account of discrimination, negative and hostile attitudes by health professionals, and difficulty with understanding and navigating health systems. ^7, 9^ This often results in delayed, restricted, or denied care. ^2, 7, 8^ Healthcare providers working within such systems are required to deliver culturally sensitive, patient-centered care despite the increased pressure for specifically tailored care. ^10, 11^ Providers report struggling with navigating language, cultural, and literacy barriers, all while trying to manage and meet the diverse and complex needs of this population. ^6, 8, 11^ Such circumstances place significant demands on health systems, which may not respond appropriately enough to address the populations’ needs, furthering health inequities and negative outcomes. ^6, 7^

Primary care plays a critical role as it often acts as the first point of contact between a system and a patient and can help address specific inequities and challenges in a whole, person-centred approach. ^8, 9^ Community-based healthcare providers are on the frontline of responding to these challenges. ^8, 9, 11^ Providing primary care to asylum seeking and refugee populations has been proven to minimise health inequalities whilst also reducing secondary care costs. ^2^ Understanding providers’ experiences is therefore key to determine how health systems support their workforces to support asylum seeking and refugee populations. ^8, 10, 11^ In view of this, improvements in the provision of specific care for these culturally diverse groups can be made.

Given the expansiveness of the topic being studied alongside the continuing evolution of current scenarios impacting staff experiences, a systematic scoping review constitutes the most appropriate methodology for achieving the intended aim of this review. This study aims to explore the experiences of healthcare providers working within primary care and community settings, when providing care to asylum seeking and refugee patients across different European Union (EU) countries.

Migration towards European countries has been consistently increasing since 2015. ^6, 12^ It is estimated that over 1.9 million individuals migrated to Europe from countries in crisis in 2020. ^13^ Based on these facts alongside the introduction of the Pact on Migration and Asylum amongst EU countries in 2026, ^14^ it was decided to geographically restrict this review to such countries only. By drawing convergences and comparisons amongst the experiences of primary care healthcare staff across different EU countries, a better understanding of key cross-border comparisons will be established.

## Methods

The Joanna Briggs Institute (JBI) guidelines were used alongside the framework for scoping reviews developed by Arksey & O’Malley as the basis of this protocol. ^15– 17^ Further details on how these methods will be followed are provided below. The following steps have been, and will be undertaken:

### 1. Identifying the research question

The main research question asks,

“ *What are the experiences of healthcare staff while providing care to asylum seekers and refugees in primary and community settings?”*

The question has been formulated using the Population-Concept-Context (PCC) framework. ^18^ The population refers to healthcare staff, the concept aligns with the provision of care for asylum seekers and refugee patients, and the context is being taken considering the population and concept existing within the primary and community care settings.

Two additional questions have been formulated:

(1) “ *What factors characterise these experiences, and why?”*, and

(2) *“Are there any differences in experiences when comparing between different EU countries?”*

The review questions were developed during meetings amongst the research team and further refined as the input and expertise of stakeholders were sought. The broad nature of the research questions will enable a thorough mapping of the body of literature pertaining to the experiences of community-based healthcare staff. All documented experiences across the various EU member states will be considered and analysed.

### 2. Identifying relevant studies

*Search Strategy:* The following electronic databases will be used to retrieve articles - PubMed, MEDLINE, CINAHL, Embase, Scopus, PsycINFO and BASE. The search engine Google Scholar will also be utilised to further identify grey literature pieces.

As per the JBI guidelines, this review is following the three steps outlined to carry out the search strategy. ^15^ A limited search has been conducted using PubMed and EBSCOhost for CINAHL, MEDLINE and PsycInfo by the research team with the help of an associate librarian at the Royal College of Surgeons in Ireland (RCSI). The results from this step are accessible in the extended data. The search terms have been chosen based on their suitability to the topic and specificity of each of the databases being used. A brief explanation of such can be found in Table 1 below. Further refinement of the search strings will follow this step. A manual search of the included studies’ references will also be carried out. This methodical search will be conducted and reported in accordance with the PRISMA-ScR criteria. ^19^

**Table 1.** Main and alternative search terms based on research question elements.

Question element	Main terms	Alternative terms
Population - Healthcare staff	Healthcare staff	Health personnel, healthcare professional, staff, nurse, nursing staff, public health nurse, GP, doctor
Concept - Experiences when caring for asylum seekers and refugees	Experience	Attitude, feeling, encounters
	Asylum seeker, refugee	International protection, protection applicant, protected person, displaced person, asylee
Context - Primary and community care	Primary care, community care	Primary healthcare, primary services, community-based care, community healthcare, community services

*Inclusion and Exclusion Criteria:* Inclusion and exclusion criteria have been established to ensure rigour through the study selection process and towards including all relevant studies in the final review. The inclusion and exclusion criteria are presented in Table 2 below. Migration can be described as ever evolving when understanding the volatility of driving effects rooted in unstable politic climates, conflict, economic insecurity, environmental stressors and familial and social relationships. To capture the experiences of primary care providers which are relevant and can be related to current scenarios, the search will be limited to literature published from 2015 onwards till the end of 2025. This is in line with the evidence available. ^6, 11– 13^ With the new EU Migrant Pact coming into full effect in mid-2026, capturing the available literature for the last decade prior to regulations around asylum applications, entitlements and supports changing will be crucial to further understand potential shifts, especially within healthcare and health systems, which may happen in the future as a result of this pact. ^14^

**Table 2.** Inclusion and exclusion criteria for study selection.

Inclusion	Exclusion
Studies about healthcare professionals working within primary and community care settings	Studies about healthcare professionals working within other settings which are not primary and community care settings; or other professionals which are not related to healthcare professions
Studies about healthcare professionals providing care to asylum seekers and/or refugees	Studies about healthcare professionals providing care to individuals who are migrants, culturally and ethnically diverse populations/individuals, but not considered as asylum seekers and/or refugees
Studies published about any of the European Union member states	Studies published about countries which are not a part of the European Union
Studies published during 2015 till the end of 2025	Studies published before 2015
Studies published in English	Studies published in another language which is not English
Original, peer-reviewed research (qualitative, quantitative, mixed methods); Review articles; Grey literature of the type of theses/dissertations, policy papers, technical reports	Grey literature pieces of the type of opinion pieces, letters to the editor, conference proceedings and abstracts, books or chapters within books

The JBI guidelines instruct researchers to sample their methods prior to executing a task to its full extent to ensure the criteria being applied is correct for the intended aim. ^15^ This validation process has been done with a sample of 10 articles retrieved from one of the intended databases. Further pilot searches will be carried out using the remaining databases. Following such, further refinements in the terms and strings being used may be required.

### 3. Study selection

Screening is divided into two activities which will be carried out using the software Covidence. ^20^ Title and abstract screening will be carried out by two independent reviewers. A third reviewer will be involved to settle disagreements between the two. Relevance of the articles being screened will mostly rely on the established inclusion and exclusion criteria. Full text reviews of articles selected after the first screening phase will be reviewed completely by the first reviewer, whilst a second reviewer will screen 25% of articles included. Here, disagreements will be settled through discussion between both reviewers, with a third reviewer being involved if needs be. The PRISMA-ScR flow chart will be used to report the activities of this step in the final review. ^19^

Similarly to previous steps, pilot screening will be done amongst reviewers to verify understanding of the set inclusion and exclusion criteria, whilst also ascertaining consistency amongst the reviewers screening decisions prior to proceeding with all screening activities. All screening activities will be done by reviewers independently.

### 4. Charting the data

Following the JBI guidelines for data extraction activities, three steps will be taken. ^15^ Firstly, a pilot phase will be done whereby two extractors will extract data independently from one another from the same five articles, utilising the data extraction sheets formulated by the research team (see extended data). This will be done to ensure consistency amongst both extractors. Data extraction of all articles will be done by the first extractor, meanwhile the second extractor will work independently to verify the data extracted is accurate and complete. Finally, all data extracted will be reviewed by both extractors to ensure consistency and completeness prior to commencing Step 5 – Data synthesis.

The data extraction process will be carried out by two reviewers, with a third reviewer being involved to settle any disagreements. All data extracted will be organised into a Microsoft Excel spreadsheet. In the spirit of ensuring all steps and decisions taken remain transparent, thorough record keeping of such will be documented.

Extraction of information will be related to outcome factors measured within each study included, documented themes regarding influences affecting experiences, and details highlighting potential facilitators and barriers. Additionally, pertinent information regarding the geographical location of the study, type of profession participants are in, and study setting shall also be extracted. The following information will be extracted from studies:

− Metadata: Author(/s), year of publication, and publication type
− Study characteristics
− Participant demographics
− Outcome(/s) measured
− Experience themes
− Reported facilitators, barriers and/or challenges
− Interventions (if any)
− Recommendations
− Limitations

By collecting this information, we will be able to identify possible similarities and differences between different countries, and professions. More details regarding all extraction criteria can be accessed through the extended data.

### 5. Collating, summarising and reporting the results

The findings will be examined, compiled, and presented using narrative and thematic analysis as according to the guidance provided by Lucas *et al*. ^21^ This method was chosen as utilising both narrative and thematic analysis proves highly beneficial to this review. Narrative synthesis will allow for the description of differences noted amongst the studies included, whereas thematic synthesis allows conclusions to be drawn out from the commonalities between studies. ^21^ In doing so, the intended purposes of the research questions are incorporated given that the research questions ask about comparisons amongst convergences and conflicting experiences documented. Additionally, the results collated will provide an overview of the extent of the research conducted in this area, alongside the gaps identified in the current research area this review falls under.

Extracted data will be presented in tables, and where necessary may be displayed graphically. Narrative descriptions will be provided to further explain the results. All data alongside supplementary materials will be made available upon publication of the review findings. This will only be done following proper engagement with stakeholders, as outlined in Step 6 below.

### 6. Consultation with relevant stakeholders

Knowledge contributors from both governmental and non-governmental organisations were contacted prior to developing this scoping review protocol. The research questions of this review have been developed based on inputs provided through these discussions.

The results from this review will be presented to key knowledge contributors prior to publication, for their interpretation of the relevance and applicability of the findings across different contexts. Furthermore, their feedback will also go to inform the recommendations put forth by this review. This is of great importance to this review, as such contributors can add more value to the findings due to their expertise.

Knowledge contributors involved are primary care providers, managers, policy makers, volunteers and community leaders. Engaged Research guidelines have been used to guide the knowledge contributor’s involvement so far and will continue to be observed. ^22^

## Implications for research and practice

The findings of this review will capture the key factors which shape primary care and community-based healthcare providers’ experiences when caring for asylum-seeking and refugee patients. Influential elements which shape both positive and negative experiences, alongside potential challenges and barriers will be outlined. By identifying information pertaining to the underlying factors effecting staff experiences, appropriate accommodations and modifications can be made by health system managers and policy makers to better protect providers’ job satisfaction, safety, and retention.

Comparisons will be made amongst different EU countries and similarly based on health profession types. Such comparisons will provide a better understanding of shared experiences and challenges, which sets the tone for better co-operation amongst different countries and emphasises the importance of health system strengthening through shared learning.

Ultimately, this review aims to inform and effect current policy, practice and training needs, on a national and international level, thereby guiding improvements in primary care, migrant health services, and better support for healthcare providers.

## Dissemination

The findings of this review will be shared in a peer-reviewed, open-access journal and presented at national and international conferences. Furthermore, the findings will be shared with knowledge contributors, key stakeholders, and other researchers who may find this information relevant.

## Conclusion

This scoping review seeks to examine the experiences of primary care health providers caring for asylum seekers and refugees, across the EU. By systematically mapping the available evidence, this review aims to uncover the challenges, opportunities, and complexities that define these frontline experiences, while laying the groundwork for meaningful comparisons across EU countries, and varied professional roles. The insights generated will offer an unprecedented window into the realities faced by health professionals, with the potential to shape policy, inform practice, and drive transformative change in the care of some of society’s most vulnerable populations.

### Study status

This review is currently on Stage 2 – Identifying relevant studies, Step 1 – Pilot search. No further steps have been taken so far to progress this review further.

## Ethical considerations

Ethical approval was not required.

## Data Availability

Reporting Guidelines: This review has been registered through Open Science Framework (OSF) ( https://osf.io/xt9bs/overview). ^23^ Extended data are available through the OSF registration. This includes:
- Pilot Searches - Data Extraction Sheet - PRISMA-ScR Checklist Data are available under the terms of the Creative Commons Attribution 4.0 International license (CC-BY-4.0).
