# Supplementary material for: Experiences of primary healthcare staff in Europe when caring for asylum seekers and refugees: A scoping review protocol
Source: HRB Open Res. 2026 Oct 6;9:38. Originally published 2026 Apr 16. [Version 2] doi: 10.12688/hrbopenres.14413.2 (PMC13687725; doi:10.12688/hrbopenres.14413.2)
Supplement: Peer review history

# Reviewer report: Reviewer response for version 2

**DOI:** 10.21956/hrbopenres.16056.r58385

**Published:** 2026-10-21

**Authors:** Stinne Glasdam

**Affiliations:** 1 Lund University, Lund, Sweden

No further comments. I wish you good luck with the work. I am looking forward to reading your final report/article.

Is the study design appropriate for the research question?

Yes

Is the rationale for, and objectives of, the study clearly described?

Partly

Are sufficient details of the methods provided to allow replication by others?

Yes

Are the datasets clearly presented in a useable and accessible format?

Yes

Reviewer Expertise:

Healthcare, sociology

I confirm that I have read this submission and believe that I have an appropriate level of expertise to confirm that it is of an acceptable scientific standard.

# Reviewer report: Reviewer response for version 2

**DOI:** 10.21956/hrbopenres.16056.r58384

**Published:** 2026-10-09

**Authors:** Daniel López-Cevallos

**Affiliations:** 1 University of Massachusetts Amherst, Amherst, Massachusetts, USA

Thank you for addressing my comments. I have no further feedback.

Is the study design appropriate for the research question?

Yes

Is the rationale for, and objectives of, the study clearly described?

Partly

Are sufficient details of the methods provided to allow replication by others?

Yes

Are the datasets clearly presented in a useable and accessible format?

Yes

Reviewer Expertise:

Public health; migration and health

I confirm that I have read this submission and believe that I have an appropriate level of expertise to confirm that it is of an acceptable scientific standard.

# Reviewer report: Reviewer response for version 1

**DOI:** 10.21956/hrbopenres.15877.r55095

**Published:** 2026-05-15

**Authors:** Daniel López-Cevallos

**Affiliations:** 1 University of Massachusetts Amherst, Amherst, Massachusetts, USA

In general this is an interesting study focusing on the experiences of primary and community healthcare providers caring for asylum seekers & refugees in Europe. While the protocol is mostly well developed, I find a few issues that authors should address before moving on to Stage 2: 

 - How many "knowledge contributors" from both governmental and non-governmental organisations were contacted?

 - Consider including some/all of these knowledge contributors as co-authors in the review. Otherwise, how are they being recognized/compensated for their time and expertise?

 - Does the project focus on the EU or European countries more broadly? In either case, why limit your search to English-language studies only, when the EU has 24 official languages?

Is the study design appropriate for the research question?

Yes

Is the rationale for, and objectives of, the study clearly described?

Partly

Are sufficient details of the methods provided to allow replication by others?

Yes

Are the datasets clearly presented in a useable and accessible format?

Yes

Reviewer Expertise:

Public health; migration and health

I confirm that I have read this submission and believe that I have an appropriate level of expertise to confirm that it is of an acceptable scientific standard, however I have significant reservations, as outlined above.

# Response

**Published:** 2026-09-21

**Authors:** Kylie Fenech

**Affiliations:** Faculty of Nursing & Midwifery, Royal College of Surgeons in Ireland, Dublin, Leinster, Ireland

Dear Dr Daniel López-Cevallos, 

 We thank you for your review of this study protocol and constructive comments provided. We appreciate the opportunity to amend and refine the protocol for this scoping review. The comments and suggestions you have provided have been considered carefully, and the appropriate revisions have been made to strengthen this manuscript. Hereunder are our responses to each comment provided. 

 Kind regards,  

 The Authors 

1. Thank you for this question. A list of potential contributors was devised, encompassing both governmental and voluntary sectors. Several meetings were held during the protocol development stage, with meetings split evenly between the two sectors, totalling ten meetings. Knowledge contributors were contacted for their input to help inform and co-design parts of the overall research project that this review falls within, with all individuals consulted so far from Ireland. Additionally, contributors from other European countries will also be contacted for their input on the results obtained from this review, and recommendations put forth from it (please see response to Reviewer 1). 
2. We appreciate this suggestion. Knowledge contributors were contacted on the premise for their input as with regards to the overall research project that this review falls within. Their general insight into the topic has provided context to the research team in co-developing and refining the research questions and aims of the entire project, in line with Engaged Research guidelines. This review follows these guidelines, with stakeholders involved within an advisory capacity. As such, a mutual agreement between the stakeholders and the research team established the involvement level of said stakeholders, which will not demand co-authorship at this point given that they have not been involved with the methodology for this review. Furthermore, this is also in line with the authorship criteria stipulated by the International Committee of Medical Journal Editors (International Committee of Medical Journal Editors, 2025). Appropriate recognition and acknowledgments will be provided further along with any research outputs from this review. Additional detail has been provided within the protocol to clarify knowledge contributors' inputs (See ‘Methods’ Section 6 – Consultation with relevant stakeholders).  
3. Thank you for these observations. These observations have already been addressed in separate comments provided by the first reviewer. This review will focus on countries which form part of the EU, EEA, and EFTA zones. Only studies published in English will be included in this review as the research team does not have the appropriate resources to consider inclusion of studies published in other languages.

We appreciate and thank you once again for your comments. We have revised the manuscript has accordingly and trust that through these amendments the manuscript and overall methods for this review have been strengthened and improved. We hope the revised version is now more suitable for publication. 

 --- 

 International Committee of Medical Journal Editors. (2025). Defining the Role of Authors and Contributors. www.icmje.org. https://www.icmje.org/recommendations/browse/roles-and-responsibilities/defining-the-role-of-authors-and-contributors.html

# Reviewer report: Reviewer response for version 1

**DOI:** 10.21956/hrbopenres.15877.r54756

**Published:** 2026-04-22

**Authors:** Stinne Glasdam

**Affiliations:** 1 Lund University, Lund, Sweden

Based on evidence that migration increases health risks for asylum seekers and refugees, while language, cultural and literacy barriers complicate care, and recognising that primary care plays a key role in addressing these health inequities, the objective of this scoping review is to explore the experiences of primary and community healthcare providers in the EU caring for these populations.

 Overall, the method is clearly described and well reflected. However, there are several aspects that could be further developed to strengthen the study:

 1. The main research question functions well. However, the additional questions are more problematic.

 (1) “What factors characterise these experiences, and why?” – I recommend revising this to: “What factors characterise these experiences, which professions experience them, and how are these experiences shaped?”

 (2) “Are there any differences in experiences when comparing between different EU countries?” – I recommend revising this to: “What similarities and differences are evident across professions and countries?”

 2. It feels somewhat limited to focus on the EU rather than Europe as a whole. There may be value in considering whether the scope could be broadened to include all of Europe as a geographic unit, to provide a more comprehensive and comparable perspective.

 3. You could add a pearl search, using Web of Sciences

 4. Table 1: The search terms appear somewhat limited. E.g. for Healthcare staff: It may be relevant to include additional keywords such as nurse assistant, laboratory technician, physiotherapist, and occupational therapist, among others. It is also important to be attentive to variations in terminology and professional titles across different countries. E.g., for Experiences: perspectives, accounts, perceptions, views, observations, relationships….

 5. Table 2: Could you justify the decision to include only European countries? Why are languages other than English excluded? And why are books or book chapters not included in this scoping review, given that a substantial amount of relevant scholarly knowledge may be located in these sources and therefore risk being overlooked?

 6. Data extraction: Could one or two additional researchers validate the extracted data to enhance reliability and reduce the risk of bias?

 7. Charting data: “By collecting this information, we will be able to identify possible similarities and differences between different countries and professions.” This is not reflected in the current research questions, which suggests that the research questions may need to be revised accordingly.

 8. Consultation with relevant stakeholders: How will you recruit stakeholders across different European countries? This section would benefit from further development and a clearer description of the recruitment strategy, including how diversity across countries and professions contexts will be ensured.

 I wish you the best of luck with your exciting study.

Is the study design appropriate for the research question?

Yes

Is the rationale for, and objectives of, the study clearly described?

Partly

Are sufficient details of the methods provided to allow replication by others?

Yes

Are the datasets clearly presented in a useable and accessible format?

Yes

Reviewer Expertise:

Healthcare, sociology

I confirm that I have read this submission and believe that I have an appropriate level of expertise to confirm that it is of an acceptable scientific standard, however I have significant reservations, as outlined above.

# Response

**Published:** 2026-09-21

**Authors:** Kylie Fenech

**Affiliations:** Faculty of Nursing & Midwifery, Royal College of Surgeons in Ireland, Dublin, Leinster, Ireland

Dear Dr Stinne Glasdam,  

 We sincerely thank you for your careful evaluation of our protocol and for the constructive comments provided. We appreciate the opportunity to revise and improve the manuscript and overall protocol for this scoping review. All comments were considered carefully, and corresponding revisions have been incorporated throughout the manuscript. Below, we provide a point-by-point response to each comment provided. 

 Kind regards, 

 The Authors 

1. Thank you for this important observation. The research questions have been revised according to your advice.
2. We appreciate the reviewer’s perspective. However, after careful consideration, we believe that maintaining the current approach is most appropriate for the objectives of this scoping review, and the larger project its findings will contribute to. Alternatively, we are opening the inclusion criteria to also include member states of the EEA and EFTA member states, thus including studies published about Iceland, Lichtenstein, Norway and Switzerland. We have clarified this aspect in the protocol and manuscript. Whilst broadening the catchment area to include all countries falling within the European continent would be beneficial, we feel that the reason for this limiter to be applied will allow us to address the review questions and aims more effectively. The rationale for this is based on the standardised consistency across the EU landscape regarding legal, economic, policy, and socio-political areas. This geographic restriction has been informed by other similar reviews, which have highlighted the importance of such restriction due to the commonality and consistency of underlying contexts (Sara et al, 2025; Stroisch et al, 2025). Furthermore, restricting such countries will allow us to draw results which reaffirm EU wide initiatives for health system strengthening, cross border learning, collaboration, and support (Sara et al, 2025; Stroisch et al, 2025).
3. Thank you for your recommendation. The protocol and manuscript have been amended to include more details regarding Step 3. A forward and backward citation search has been done on all included studies, through Citation Chaser.
4. Thank you for this important observation. The search terms have been chosen and revised according to each specific database being used, and where necessary, search strings have been broadened to include other terms. These were further refined and finalised with assistance from a library expert in our university, responsible for developing search terms for various literature review studies. In doing so, the final search strings used have been amended to the best possible way to include additional alternative search terms, to further maximise the number of hits and without jeopardising the sensitive quality of the results retrieved. The search strings used will be made available alongside all other pertinent information through the protocol registration on Open Science Framework.
5. Thank you for the important observations made on the limiters applied to this review, and we appreciate these valuable suggestions. The review is being restricted to countries as part of the EU, EEA, and EFTA zones due to the commonality of socio-political underpinnings, alongside commonalities across economic and policy areas. Only studies published in English will be included in this review as we do not have the facility nor the capacity to include studies published in other languages. Given the sensitivity of the nature of this topic and the depth of information that will be reviewed, the research team made the executive decision that publications such as books and chapters within books would be insufficient for the level of data being required, and somewhat outside the overall objectives of the proposed scoping review.  
6. We appreciate the reviewer drawing attention to this limitation. This was an important point that had not been sufficiently addressed in the original protocol. We have now revised this to provide a clearer explanation. Two researchers will be extracting data from the included studies, whilst an additional researcher will be involved in settling disagreements and reaching consensus, where required. 
7. Thank you for this comment. This has already been further elaborated on above in a separate comment. The research questions have been revised accordingly to better fit the review objectives. 
8. Thank you for this important observation. We fully agree that additional detail regarding such would improve the quality of this review in general. We have revised the protocol and manuscript accordingly. We provided further explanation in the ‘Methods’ section (Section 6 – Consultation with relevant stakeholders). European stakeholders will be contacted based on their expertise and relevance to the review topic. Individuals working within primary care and refugee care will be contacted based on their expertise, professional background, and based on recommendations from other stakeholders on who else should be involved. Whilst there is no prescriptive strategy being used with recruitment for stakeholder engagement, this will become clearer as the review progresses and will determine who should be involved.

We again thank you for your constructive feedback and believe that the revisions undertaken have improved the clarity, rigour, and transparency of the protocol and overall methods of this review. We hope the revised version is now more suitable for publication. 

 --- 

 Sara, M. G., Jocelyn, S., & Sébastien, C. (2025). Advanced practice nursing in the European Union: A scoping review. Nursing Outlook, 73(6), 102588. https://doi.org/10.1016/j.outlook.2025.102588 

 Stroisch, S., Angelini, V., Schnettler, S., & Vogt, T. (2025). Health Outcomes in EU Cross-Border Regions: A Scoping Review. Public health reviews, 46, 1608170. https://doi.org/10.3389/phrs.2025.1608170
